# Nuclear magnetic resonance‐based metabolomics identifies phenylalanine as a novel predictor of incident heart failure hospitalisation: results from PROSPER and FINRISK 1997

**DOI:** 10.1002/ejhf.1076

**Published:** 2017-12-11

**Authors:** Christian Delles, Naomi J. Rankin, Charles Boachie, Alex McConnachie, Ian Ford, Antti Kangas, Pasi Soininen, Stella Trompet, Simon P. Mooijaart, J. Wouter Jukema, Faiez Zannad, Mika Ala‐Korpela, Veikko Salomaa, Aki S. Havulinna, Paul Welsh, Peter Würtz, Naveed Sattar

**Affiliations:** ^1^ Institute of Cardiovascular and Medical Sciences (ICAMS), BHF Glasgow Cardiovascular Research Centre University of Glasgow Glasgow UK; ^2^ Glasgow Polyomics, Joseph Black Building University of Glasgow Glasgow UK; ^3^ Robertson Centre for Biostatistics, Boyd Orr Building University of Glasgow Glasgow UK; ^4^ Computational Medicine, Faculty of Medicine and Biocenter Oulu University of Oulu Oulu Finland; ^5^ NMR Metabolomics Laboratory, School of Pharmacy University of Eastern Finland Kuopio Finland; ^6^ Leiden University Medical Centre Leiden The Netherlands; ^7^ Inserm Centre d'Investigation Clinique (CIC) 1443 Université de Lorraine Lorraine France; ^8^ Centre Hospitalier Régional Universitaire (CHRU) de Nancy Nancy France; ^9^ Medical Research Council Integrative Epidemiology Unit University of Bristol Bristol UK; ^10^ Population Health Science, Bristol Medical School University of Bristol Bristol UK; ^11^ Systems Epidemiology Baker Heart and Diabetes Institute Melbourne Victoria Australia; ^12^ Department of Epidemiology and Preventive Medicine, School of Public Health and Preventive Medicine, Faculty of Medicine, Nursing and Health Sciences, The Alfred Hospital Monash University Melbourne Victoria Australia; ^13^ National Institute for Health and Welfare (THL) Helsinki Finland; ^14^ Institute for Molecular Medicine (FIMM) University of Helsinki Helsinki Finland; ^15^ Research Programs Unit, Diabetes and Obesity University of Helsinki Helsinki Finland

**Keywords:** Metabolomics, Advanced lipoprotein profiling, Heart failure, PROSPER, Phenylalanine, FINRISK

## Abstract

**Aims:**

We investigated the association between quantified metabolite, lipid and lipoprotein measures and incident heart failure hospitalisation (HFH) in the elderly, and examined whether circulating metabolic measures improve HFH prediction.

**Methods and results:**

Overall, 80 metabolic measures from the PROspective Study of Pravastatin in the Elderly at Risk (PROSPER) trial were measured by proton nuclear magnetic resonance spectroscopy (n = 5341; 182 HFH events during 2.7‐year follow‐up). We repeated the work in FINRISK 1997 (n = 7330; 133 HFH events during 5‐year follow‐up). In PROSPER, the circulating concentrations of 13 metabolic measures were found to be significantly different in those who were later hospitalised for heart failure after correction for multiple comparisons. These included creatinine, phenylalanine, glycoprotein acetyls, 3‐hydroxybutyrate, and various high‐density lipoprotein measures. In Cox models, two metabolites were associated with risk of HFH after adjustment for clinical risk factors and N‐terminal pro‐B‐type natriuretic peptide (NT‐proBNP): phenylalanine [hazard ratio (HR) 1.29, 95% confidence interval (CI) 1.10–1.53; P = 0.002] and acetate (HR 0.81, 95% CI 0.68–0.98; P = 0.026). Both were retained in the final model after backward elimination. Compared to a model with established risk factors and NT‐proBNP, this model did not improve the C‐index but did improve the overall continuous net reclassification index (NRI 0.21; 95% CI 0.06–0.35; P = 0.007) due to improvement in classification of non‐cases (NRI 0.14; 95% CI 0.12–0.17; P < 0.001). Phenylalanine was replicated as a predictor of HFH in FINRISK 1997 (HR 1.23, 95% CI 1.03–1.48; P = 0.023).

**Conclusion:**

Our findings identify phenylalanine as a novel predictor of incident HFH, although prediction gains are low. Further mechanistic studies appear warranted.

## Introduction

The prevention of heart failure (HF) is an important clinical issue. Patients with HF have a high mortality and impaired quality of life, so identifying those at risk is important.^1^, ^2^ The risk of HF increases with age but typical symptoms of HF, such as shortness of breath, may be absent in the elderly (or masked by other co‐morbidities); the prognosis of HF is poor, and the mechanisms of HF differ in the elderly.^3^ Treatment of hypertension and dyslipidaemia, prevention of diabetes, smoking cessation, increased exercise, weight reduction, and reduced alcohol intake have been associated with lower risks for HF.^4^–^6^ At present, symptomatic patients have B‐type natriuretic peptide (BNP) or N‐terminal proBNP (NT‐proBNP) concentrations measured as a rule‐out test for HF, a cost‐effective strategy to increase the positive predictive value of echocardiography. However, routine screening for this marker as part of cardiovascular disease (CVD) risk screening is not cost‐effective as the assay is currently much more expensive than other routine clinical laboratory tests.^7^ More effective screening for prevalent HF, perhaps using ‘omics technologies, in combination with more effective interventions, has been described as an urgent need in the HF clinical arena.^7^ Such strategies might help pave the way toward better identification of HF, or identify novel treatment strategies. Studies to improve the understanding of HF aetiology and generate new hypothesis, particularly in the elderly, are also needed.

Metabolomics is the study of the small molecule complement of a system using a variety of methods, mainly mass spectrometry (MS) and proton nuclear magnetic resonance (^1^H‐NMR) spectroscopy.^8^ Both methods are complementary, each with their own strengths and limitations.^8^ MS metabolomics methods are generally very sensitive, detecting thousands of metabolites, but routinely provide only relative (rather than absolute) quantitation. Generally, ^1^H‐NMR metabolomics methods have poor sensitivity in comparison to MS but do provide absolute quantitation, higher throughput (resulting in reduced costs), and better reproducibility. Metabolomics is of particular interest, since HF is strongly linked to metabolic dysfunction. Dysregulation of cardiac energy metabolism and cardiac remodelling are key features of HF that may result in changes in circulating metabolite concentrations, and adverse metabolic states like diabetes increase HF risk.^9^–^11^ Whether changes in metabolic profile precede incident HF is therefore an important mechanistic line of research. Metabolomics has been used to study prevalent HF,^9^, ^10^, ^12^–^20^ but most studies have been cross‐sectional in nature. Presently, only one study has prospectively investigated the association of the metabolome with future HF risk.^13^ This study used an untargeted gas chromatography/MS metabolomics method to identify two metabolites associated with incident HF.^13^ In contrast, we here employ a ^1^H‐NMR spectroscopy method that allowed detailed lipoprotein subclass analysis, in addition to small molecule and lipid concentrations,^21^ to study samples from the PROspective Study of Pravastatin in the Elderly at Risk (PROSPER) trial.^22^ We hypothesised that metabolites, lipids and lipoproteins would associate with HF hospitalisation (HFH) in elderly men and women and improve HFH prediction beyond established clinical predictors and NT‐proBNP.

## Methods

### Study cohort: PROSPER

The PROSPER trial design has been published.^22^ In brief, this was a double‐blind, randomised, placebo‐controlled trial investigating the benefit of pravastatin (40 mg/day) in elderly individuals at risk of CVD. Participants were identified in the primary care setting from three centres: Glasgow, Scotland; Cork, Ireland; and Leiden, The Netherlands. Overall, 5804 elderly adults (70–82 years old) were enrolled. All participants had high‐normal to high cholesterol concentrations (4.0–9.0 mmol/L) at baseline. Additionally, 50% of patients had evidence of vascular disease (physician diagnosed stable angina, stroke, transient ischaemic attack, or myocardial infarction) and the remaining 50% of patients had high risk of vascular disease as they had either hypertension, diabetes, or were smokers. Individuals with congestive HF [New York Heart Association (NYHA) class III and IV] were excluded. The primary outcome measure of PROSPER was a composite CVD outcome. In the current study, the endpoint of interest was hospitalisation for incident HF. This was defined based on a combination of symptoms (e.g. shortness of breath) and signs, including chest radiograph with fluid congestion or echocardiogram with severely diminished left ventricular function.^23^ Patients were recruited between December 1997 and May 1999, and the mean follow‐up period was 3.2 years.^24^ The investigation conforms with the principles outlined in the Declaration of Helsinki. The institutional ethics review boards of all three European centres approved the study protocol.^25^ All participants provided written informed consent to participate in the study and for long‐term follow‐up.

**Figure 1:**
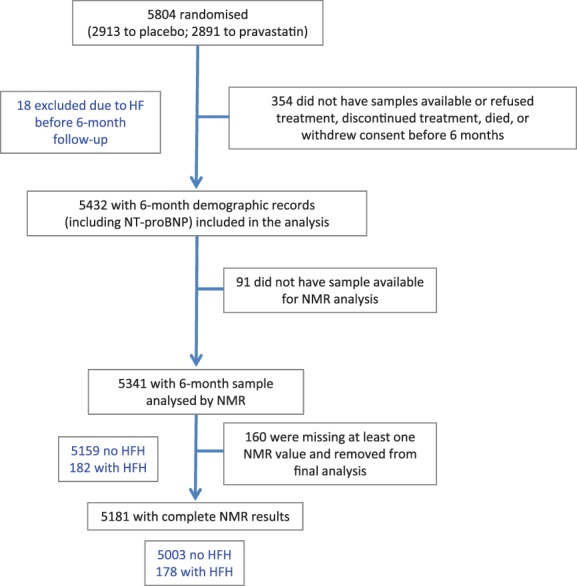
Flow diagram of the PROSPER study. Of the 5432 samples with 6‐month demographic records, including N‐terminal pro‐B‐type natriuretic peptide (NT‐proBNP), 5341 had 6‐month sample analysed by nuclear magnetic resonance (NMR) spectroscopy. Of these samples, 182 were from individuals who were later hospitalised for heart failure (HF). 160 samples were excluded due to missing metabolic measures. Of these 5181, 178 were later hospitalised for HF. HFH, heart failure hospitalisation.

Fasting venous blood samples were collected at baseline and at 3‐month intervals and biobanked at –80 °C. For the present study, previously unthawed 6‐month post‐randomisation samples were used, employing the study as a cohort study and adjusting for randomised treatment in analysis. Overall, 5341 samples were available for this study, having sample available for ^1^H‐NMR analysis and available 6‐month NT‐proBNP and other measurements^24^ (*Figure* 1). Estimated glomerular filtration rate (eGFR) was calculated based on routinely available creatinine, using the Modification of Diet in Renal Disease (MDRD) equation.^26^ Eighteen participants who had died or experienced HFH in the first 6 months of follow‐up were excluded from the analysis since 6‐month samples were used as predictors of incident HFH.

### External replication cohort: FINRISK

The population‐based FINRISK 1997 cohort was used as an external replication cohort. Participants were aged between 25 and 74 years at recruitment and were derived from the general population in five study areas across Finland.^27^ All participants provided written informed consent and the study protocol was approved by the local ethics committees. A total of 8444 individuals were recruited and NMR metabolic measures were available for 7602 baseline serum samples. Semi‐fasting venous blood samples were biobanked at –80 °C. For the present study, samples with only one previous freeze–thaw cycle were used. Incident HF during follow‐up was identified through the Finnish National Hospital Discharge Register and Cause‐of‐Death Register using the International Classification of Diseases diagnosis codes, 10th revision. Additionally, nationwide drug reimbursement and prescription registers were used to identify individuals on HF medication. This method of registry follow‐up has been validated.^27^ We curtailed follow‐up to 5 years (longer than the 2.7 years in PROSPER) since this is a younger, healthier, cohort. Any individual with prior HF was excluded, leaving 7330 individuals included in this study. Of these, 133 individuals were classed as having an incident HF event within 5 years of follow‐up. Estimated GFR was calculated using NMR measured creatinine concentrations and the MDRD equation as above.

### Metabolite, lipid and lipoprotein quantification

Circulating metabolic measures were quantified using high‐throughput serum NMR metabolomics (Brainshake Ltd, Helsinki, Finland) as previously described.^21^, ^28^, ^29^ The quantified metabolite measures are as described by Soininen et al. 21 This includes various metabolites (e.g. amino acids and creatinine) and extracted lipids (e.g. sphingomyelin and omega‐6 fatty acids). The lipoprotein measures include particle concentrations for 14 lipoprotein subclasses, as well as their lipid (total lipid, free cholesterol, cholesterol ester, phospholipid, and triglyceride) content. A total of 233 measures were reported. Only 80 metabolite, lipid or lipoprotein measures were included in the analysis since the majority of the lipoprotein measures were excluded due to redundancy (overlapping nature) of many of the lipoprotein measures. Ratios were also excluded, with the exception of the fatty acids where normalisation to total fatty acids allows more meaningful biological interpretation. The same NMR platform was used for metabolic profiling of serum samples from the FINRISK 1997 cohort.

### Statistical analysis

All metabolite concentrations were log transformed prior to analysis to obtain approximately normal distributions. The metabolite measures were subsequently centred and scaled to standard deviation (SD) units.

Mean imputation of missing continuous baseline characteristics (e.g. eGFR or total cholesterol) was carried out. For categorical variables either 3‐month or baseline values replaced the missing 6‐month values. Measured metabolite or lipoprotein concentrations denoted zero, as reported by NMR spectroscopy, were imputed as half of the minimum reported value. All observations with any NMR measure reported as ‘not available’ were excluded from the analysis of hazard ratios (HR) and later statistical analysis.

An analysis comparing metabolite or lipoprotein particle concentrations between participants, split by HFH outcome status, was carried out using the t‐test, and the method of Benjamini and Hochberg was used to control for multiple testing.^30^ P‐values were adjusted using a false discovery rate (q) of 0.1, raw P‐values of ≤0.014 were considered significant.

Survival analysis was carried out using Cox proportional hazards regression and the proportional hazards assumption was verified by the inclusion of time‐dependent covariates in the model. Metabolite and lipoprotein associations were adjusted for treatment group, age, sex, smoking status, country, body mass index (BMI), systolic blood pressure (SBP), diastolic blood pressure (DBP), eGFR, NT‐proBNP, history of myocardial infarction, coronary artery bypass graft, percutaneous transluminal coronary angioplasty, transient ischaemic attack, stroke, diabetes mellitus, angina, claudication, peripheral vascular disease, treatment with angiotensin‐converting enzyme (ACE) inhibitors, beta‐blockers, calcium channel blockers, anti‐arrhythmic drugs, and/or diuretics. Medications are as recorded at baseline (first visit) whereas all other measures were as recorded at 6 months. A competing risk model was also performed, which did not alter the HRs obtained. In the FINRISK 1997 cohort, Cox models were adjusted for sex, prior major coronary event (including CVD and revascularisation), BMI, SBP, DBP, NT‐proBNP, smoking, diabetes mellitus, lipid‐lowering and blood pressure‐lowering therapy. Age was used as the time‐scale.

For risk prediction analysis in PROSPER, NMR‐derived metabolic measures associated with HFH at the P ≤ 0.1 significance level were re‐introduced into a model containing classical risk factors using a backward selection method to identify the metabolites independently associated with HF.

The established risk factors (Model 1) and the established risk factors with significant metabolite/lipoprotein measures (Model 2) were then used in risk prediction analysis by comparing the 2.7‐year predictive risk of HFH between the two models. The C‐index and 95% confidence intervals (CIs) were calculated, using bootstrapping for 1000 runs for both point estimates and 95% CIs. The gain in predictive ability of Model 2 over Model 1 was calculated using bootstrapping. Net reclassification comparing Model 2 with Model 1 was also assessed using both continuous and categorical risk intervals of <2.5%, ≥2.5 to <5%, and ≥5%.

## Results

### Baseline characteristics in PROSPER

In PROSPER, 182 participants out of 5341 were hospitalised with incident HF during follow‐up (3.4%) (Figure 1). Patients hospitalised with incident HF were older; more likely to be male; more likely to be recruited in Scotland or Ireland; more likely to have had a previous myocardial infarction, angina, or claudication; more likely to have been on ACE inhibitors or anti‐arrhythmic therapy at baseline; and had lower eGFR, lower DBP, and raised NT‐proBNP (Table 1).

**Table 1:** Demographic characteristics: incident heart failure hospitalisation (HFH) vs. no HFH in PROSPER

Characteristic	No HFH (n = 5159)	HFH (n = 182)	P‐value
Age, years	75.78 ± 3.34	76.66 ± 3.55	**<0.001**
Male sex	2480 (48.1)	108 (59.3)	**0.003**
Current smoker	1289 (25.0)	43 (23.8)	0.70
Country			
Scotland	2217 (43.0)	88 (48.4)	**0.004**
Ireland	1920 (37.2)	76 (41.8)	
The Netherlands	1022 (19.8)	18 (9.9)	
BMI, kg/m^2^	26.57 ± 4.18	27.05 ± 4.16	0.10
Myocardial infarction	705 (13.7)	53 (29.3)	**<0.001**
SBP	154.97 ± 21.95	155.22 ± 22.78	0.67
DBP	85.02 ± 11.01	82.44 ± 11.56	**0.005**
CABG	141 (2.7)	4 (2.2)	0.66
PTCA	93 (1.8)	3 (1.6)	0.88
TIA	400 (7.8)	16 (8.8)	0.61
Stroke	202 (3.9)	12 (6.6)	0.07
Angina	1289 (25.0)	80 (44.0)	**<0.001**
Claudication	334 (6.5)	26 (14.3)	**<0.001**
PVD surgery	107 (2.1)	5 (2.7)	0.53
ACE inhibitors	920 (17.8)	59 (30.8)	**<0.001**
Beta‐blockers	1333 (25.8)	40 (22.0)	0.24
Calcium channel blockers	1286 (24.9)	56 (30.8)	0.07
Anti‐arrhythmics	124 (2.4)	10 (5.5)	**0.009**
Diuretics	2060 (39.9)	85 (46.7)	0.07
Diabetes	360 (7.0)	18 (9.9)	0.13
eGFR, mL/min/1.73 m^2^	60.36 ± 14.55	56.63 ± 15.37	**<0.001**
Treatment group (pravastatin)	2564 (49.7)	85 (47.6)	0.43
6‐month NT‐proBNP, ng/L	143.1 (77.9–274.7)	522.9 (215.8–1144.0)	**<0.001**

Demographic characteristics are detailed for 5341 individuals with metabolic measures quantified by nuclear magnetic resonance spectroscopy. Baseline (or 6‐month for NT‐proBNP) summary characteristics are reported as means ± standard deviation for continuous measures, with the exception of NT‐proBNP concentration which was not normally distributed (median, IQR), and as numbers with percentage for categorical variables. Measures that are significantly different between those later hospitalised for HF vs. those who were not are shown in bold (P < 0.05). Any missing data at 6 months was imputed from 0 months.

ACE, angiotensin‐converting enzyme; BMI, body mass index; CABG, coronary artery bypass graft; DBP, diastolic blood pressure; eGFR, estimated glomerular filtration rate; HF, heart failure; NT‐proBNP, N‐terminal pro‐B‐type natriuretic peptide; PTCA, percutaneous transluminal coronary angioplasty; PVD, peripheral vascular disease; SBP, systolic blood pressure; TIA, transient ischaemic attack.

### Associations of metabolites and lipoprotein measures with heart failure in PROSPER

Eighteen out of 80 metabolic measures (as quantified by NMR) were associated with HFH, with P‐values of <0.05. After correction for false discovery rate (Benjamini and Hochberg method), 13 measures were considered statistically significant (raw P‐value of ≤0.014). Distributions of the concentrations of the metabolites and lipoprotein measures stratified by HFH status are shown in Table 2 for measures with raw P‐values of <0.05 and in the supplementary material online, Table S1, for measures with raw P‐values of >0.05. Three of the markers associated with HFH were small molecules: phenylalanine, creatinine, and 3‐hydroxybutyrate; and one was a measure of glycoprotein acetyls, from highly glycosylated acute phase proteins.^31^ All four measures were positively associated with HFH. Nine were lipoprotein related measures such as mean low‐density lipoprotein (LDL) diameter: all were negatively associated with HFH. Of these, six were specifically related to high‐density lipoprotein (HDL) particles (apolipoprotein A1), concentration of medium HDL particles, concentration of small HDL particles, total cholesterol content of HDL, and phospholipid content of HDL. Routine lipid measures such as LDL‐cholesterol or HDL‐cholesterol were not associated with incident HFH risk.^23^, ^32^

**Table 2:** Metabolic measures quantified by nuclear magnetic resonance metabolomics in heart failure hospitalisation (HFH) vs. no HFH in PROSPER

Metabolite or lipoprotein measure	No HFH (n = 5159)	HFH (n = 182)	P‐value
Apolipoprotein A1 (g/L)	1.54 (1.44–1.65)	1.49 (1.40–1.60)	**<0.001**
Concentration of medium HDL particles (nmol/L)	1.77 (1.59–1.97)	1.64 (1.48–1.88)	**<0.001**
Concentration of small HDL particles (nmol/L)	4.40 (4.17–4.66)	4.30 (3.99–4.58)	**<0.001**
Creatinine (μmol/L)	71.00 (60.50–82.90)	76.70 (64.10–96.10)	**<0.001**
Glycoprotein acetyls (mmol/L)	1.27 (1.18–1.38)	1.32 (1.22–1.42)	**<0.001**
Phenylalanine (mmol/L)	45.10 (40.70–49.80)	47.85 (43.30–52.40)	**<0.001**
Phospholipids in HDL (μmol/L)	1.32 (1.16–1.52)	1.25 (1.10–1.44)	**<0.001**
Esterified cholesterol (%)	71.35 (70.05–72.55)	70.94 (69.44–72.00)	**0.001**
Mean diameter for LDL particles (nm)	23.70 (23.60–23.70)	23.70 (23.60–23.70)	**0.002**
Total cholesterol in HDL2 (mmol/L)	0.83 (0.68–1.03)	0.76 (0.64–0.95)	**0.003**
Total cholesterol in HDL (mmol/L)	1.31 (1.14–1.50)	1.24 (1.10–1.44)	**0.004**
3‐Hydroxybutyrate (mmol/L)	0.10 (0.07–0.15)	0.12 (0.08–0.17)	**0.011**
Total phospholipids (μmol/L)	2.65 (2.41–2.90)	2.59 (2.34–2.87)	**0.014**
Citrate (μmol/l)	96.80 (81.40–112.00)	99.85 (85.10–117.00)	0.032
Ratio of omega‐3 fatty acids to total fatty acids	3.81 (3.35–4.42)	3.64 (3.29–4.23)	0.033
Concentration of large HDL particles (nmol/L)	1.00 (0.76–1.29)	0.92 (0.70–1.21)	0.034
Lactate (mmol/L)	2.33 (1.80–3.39)	2.44 (1.94–3.84)	0.04
Acetate (mmol/L)	0.03 (0.02–0.03)	0.02 (0.02–0.03)	0.046

Values are expressed as median and interquartile range. Only metabolite and lipoprotein measures that are significantly different between HFH vs. no HFH are shown (P < 0.05) (see supplementary material online, Table S1, for metabolite and lipoprotein measures not significantly different between HFH and no HFH). Measures shown in order of ascending P‐value; those ≤0.014 are shown in bold, notionally significant after correcting for false discovery using the Benjamini and Hochberg method and a false discovery rate of 0.1.

HDL, high‐density lipoprotein; LDL, low‐density lipoprotein.

**Figure 2:**
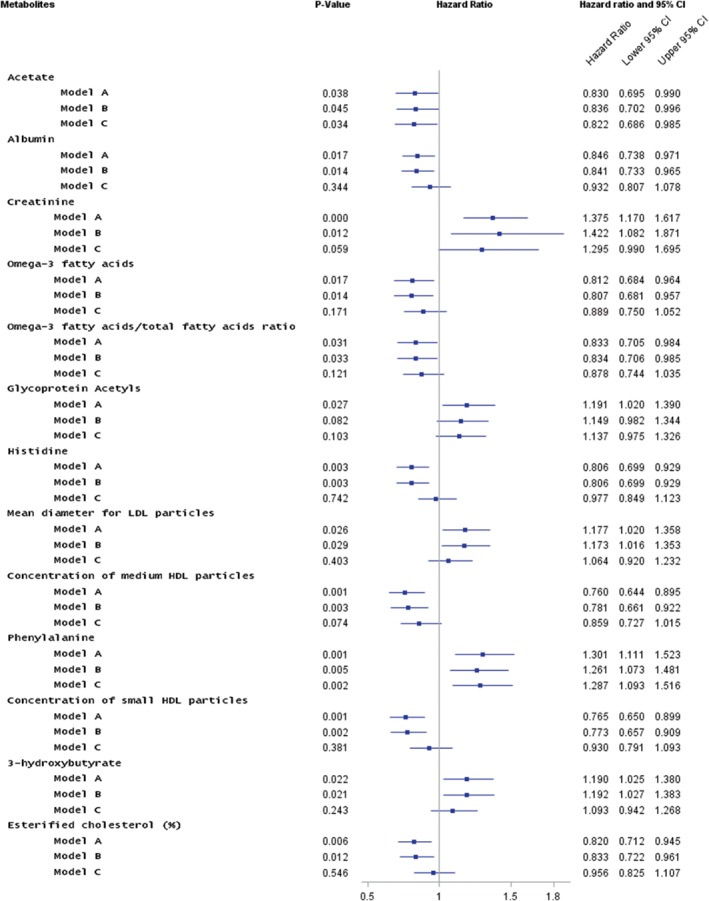
Forest plot of hazard ratios and 95% confidence intervals (CI) of association for metabolites with incident heart failure hospitalisation (HFH) in PROSPER during 2.7 years of follow‐up. Associations were adjusted for treatment group, age, sex, smoking status, country, body mass index (BMI), myocardial infarction, systolic (SBP) and diastolic blood pressure (DBP), coronary artery bypass graft, percutaneous transluminal coronary angioplasty, transient ischaemic attack, stroke, angina, claudication, peripheral vascular disease, diabetes, estimated glomerular filtration rate (eGFR), N‐terminal pro‐B‐type natriuretic peptide (NT‐proBNP) concentration (6‐month) and treatment with angiotensin‐converting enzyme inhibitors, beta‐blockers, calcium channel blockers, anti‐arrhythmics and diuretics (note all medications are as recorded at baseline, 0 month). Nuclear magnetic resonance measures with P < 0.05 in Model A are shown. HDL, high‐density lipoprotein; LDL, low‐density lipoprotein. Model A: adjusted for sex, BMI, SBP, DBP, current smoking, diabetes, pravastatin/placebo, blood pressure‐lowering therapy, major coronary events/baseline cardiovascular disease. Model B: adjusted as for Model A plus eGFR. Model C: adjusted as for Model B plus NT‐proBNP.

In multivariable models adjusting for classical risk factors and NT‐proBNP, phenylalanine had the strongest association with HFH (HR 1.29 for one SD higher phenylalanine level, 95% CI 1.10–1.53; P = 0.002) (Figure 2 shows measures with P < 0.05; the supplementary material online, Table S2, for all). Acetate was inversely associated with HFH (HR 0.81 for one SD higher acetate, 95% CI 0.68–0.98; P = 0.026). A competing risk model was also performed, which did not alter the HRs obtained.

### Multivariable models for risk prediction

In a backward selection procedure (Model 2, including established risk factors), phenylalanine and acetate were retained as significant predictors. Model 2 was compared to Model 1 (established risk factors only; *Table* 3). Model 1, which includes NT‐proBNP, has a fair‐to‐good predictive ability (C‐index 0.785, 95% CI 0.754–0.816). The addition of phenylalanine and acetate (Model 2) did not significantly improve the predictive ability (C‐index 0.787, 95% CI 0.758–0.819; change in C‐index 0.0036, 95% CI –0.046 to 0.051; *P* = 0.880). However, the net reclassification index (NRI) demonstrated that Model 2 improved classification of participants who did not experience HFH to more appropriate risk categories in both categorical and continuous models (categorical NRI for non‐cases 0.010, 95% CI 0.001–0.019; *P* = 0.012; continuous NRI for non‐cases 0.143, 95% CI 0.115–0.170; *P* < 0.001). Only the overall continuous NRI showed an improvement with Model 2 compared to Model 1 (0.205, 95% CI 0.055–0.355; *P* = 0.007).

**Table 3:** Risk prediction metric values for incident heart failure hospitalisation in PROSPER

	Metric point estimate (95% CI)	*P*‐value
Established risk factors (Model 1)		
C‐index (95% CI)	0.785 (0.754–0.816)	
Established risk factors plus phenylalanine and acetate (Model 2)		
C‐index (95% CI)	0.787 (0.758–0.819)	
Gain in predictive ability in Model 2 vs. 1		
C‐index change	0.0036 (–0.0457 to 0.0505)	0.880
Categorical NRI of 3‐year risk		
Cases	0.0000 (–0.0470 to 0.0470)	0.500
Non‐cases	0.0104 (0.0014–0.0194)	**0.012**
Overall	0.0104 (–0.0374 to 0.0582)	0.670
Continuous NRI of 3‐year risk		
Cases	0.0621 (–0.0849 to 0.2092)	0.408
Non‐cases	0.1429 (0.1155–0.1703)	**<0.001**
Overall	0.2051 (0.0555–0.3546)	**0.007**

Model 1 (established risk factors, including NT‐proBNP) and Model 2 (established risk factors plus phenylalanine and acetate) have a fair‐to‐good C‐index value. Adding phenylalanine and acetate to usual risk factors (including NT‐proBNP) did not significantly improve the C‐index. It did improve prediction of non‐cases (statistically significant improvement in categorical and continuous NRI).

CI, confidence interval; NRI, net reclassification index.

### External replication: FINRISK 1997 cohort

**Figure 3:**
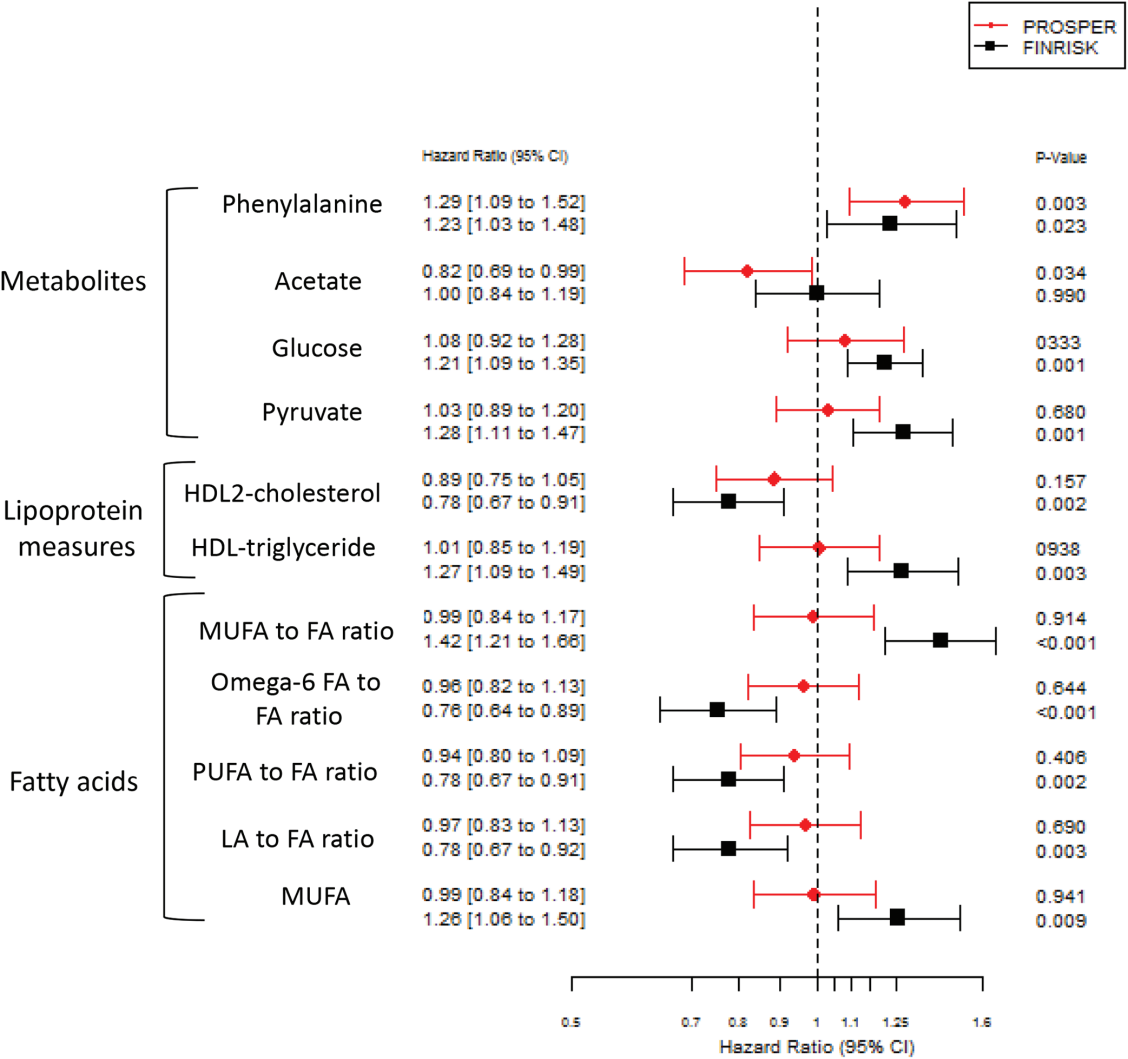
Comparison of hazard ratios for incident heart failure hospitalisation or heart failure events in PROSPER and the FINRISK 1997 cohort. Hazard ratios are adjusted for age, sex, body mass index, systolic and diastolic blood pressure, current smoking, diabetes, pravastatin/placebo (in PROSPER); blood pressure‐lowering therapy, major coronary events/baseline cardiovascular disease; estimated glomerular filtration rate and N‐terminal pro‐B‐type natriuretic peptide. Only metabolic measures with P < 0.01 in at least one cohort are displayed (with the exception of acetate). CI, confidence interval; FA, fatty acid; HDL, high‐density lipoprotein; LA, linoleic acid; MUFA, monounsaturated fatty acid; PUFA, polyunsaturated fatty acid.

In the FINRISK 1997 cohort, 24 out of 85 metabolic measures (as quantified by NMR metabolomics) were significantly associated with 5‐year incident HF risk (*P* < 0.05) (supplementary material online, *Table S3*). Six measures were inversely associated with HF [ratio of omega‐6 fatty acids to total fatty acids, ratio of polyunsaturated fatty acids (PUFA) to total fatty acids, HDL2‐cholesterol, ratio of linoleic acid to total fatty acids, 3‐hydroxybutyrate and LDL‐cholesterol), and 18 were positively associated with HF [monounsaturated fatty acid (MUFA) to total fatty acid ratio, pyruvate, HDL‐triglycerides, isoleucine, total triglycerides, MUFA, tyrosine, mean very low‐density lipoprotein (VLDL) diameter, phenylalanine, lactate, alanine, glycoprotein acetyls, glucose, VLDL‐triglyceride, triglyceride ratio to phosphoglycerides, intermediate density lipoprotein (IDL)‐triglyceride, LDL‐triglyceride, and IDL‐phospholipid concentration]. The strongest predictor of HF in PROSPER, phenylalanine, was replicated in FINRISK (HR 1.23 for one SD increase, 95% CI 1.03–1.48; *P* = 0.023). The association of acetate was not replicated (HR 1.0, 95% CI 0.842–1.19; *P* = 0.99). Estimated HRs were visually compared between PROSPER and FINRISK (*Figure* 3). This comparison demonstrates that HRs for a number of other metabolites for incident HF appear stronger in a middle‐aged cohort (derived from the general population) compared to an elderly cohort (recruited with pre‐existing CVD or at high risk of CVD^22^).

## Discussion

We report that in PROSPER, 13 metabolites and lipoprotein measures were associated with HFH but only phenylalanine (positively) and acetate (inversely) were independently associated with HFH after adjustment for classical risk factors inclusive of NT‐proBNP. These two metabolites only moderately improved prediction of HFH (no significant improvement in C‐index) with slight improvement in the net reclassification on non‐cases (i.e. improved specificity). However, the pathways underlying the metabolite associations with HFH are arguably more interesting, suggesting novel insights into the development of HF, which is known to be mechanistically entwined with functional metabolic changes, such as glycolysis, gluconeogenesis and lipolysis.^11^ The improvement in overall continuous NRI, especially net reclassification of non‐cases, is potentially important. The final model correctly down‐classified non‐cases, potentially reducing the burden on echocardiography facilities and increasing the positive value of echocardiography without a statistically significant net reclassification of cases (no significant increase in the number of cases not referred for echocardiography, i.e. missed).

In this study, of the multiple metabolites measured in the elderly, phenylalanine had the strongest association with incident HFH (HR 1.29 per log SD increase, 95% CI 1.10–1.53; *P* = 0.002). Notably, raised phenylalanine concentration has been identified in cross‐sectional studies comparing individuals with established HF to normal controls.^12^, ^15^, ^17^, ^33^, ^34^ However, to our knowledge, ours is the first study to suggest phenylalanine also predicts incident HFH. A similar association was observed in the external replication cohort, despite their substantial differences in age (FINRISK population being much younger) and co‐morbidities (far fewer in FINRISK), and consequently different HF risk profiles. The consistency in this association is therefore useful to see, particularly given that risk factor associations with adverse outcomes, such as HF, typically weaken in older cohorts (as observed for blood pressure,^35^ adiponectin,^36^ and cholesterol^23^, ^37^). Phenylalanine is an essential amino acid and a precursor for tyrosine. The ability of increased phenylalanine to predict incident HFH may indicate several potential mechanisms. Firstly, it may reflect early altered protein catabolism.^10^, ^12^, ^33^ Increased phenylalanine may originate from both cardiac and/or skeletal muscles, perhaps due to shared underlying pathogenesis or skeletal muscle degradation resulting from reduced tissue blood supply in individuals with HF.^17^ Increased protein catabolism may also be a result of insulin resistance, a putative risk factor for HF.^27^, ^33^ Secondly, increased phenylalanine concentrations may reflect impaired uptake and utilisation of amino acids,^15^ impaired renal function,^38^ or impaired liver function with decreased phenylalanine hydroxylation.^12^ Thirdly, they may indicate depletion of tetrahydrobiopterin (a co‐factor for phenylalanine hydroxylase which converts phenylalanine to tyrosine), resulting from induction of nitric oxide synthase‐2, for which tetrahydrobiopterin is also a co‐factor.^12^ Finally, it is known that phenylalanine and tyrosine are precursors of catecholamines, including adrenaline and noradrenaline, and higher concentrations are observed in HF due to stress response to reduced cardiac output.^33^ Clearly, further mechanistic studies are needed to determine why phenylalanine is predictive for HF and whether this association could potentially be causal in nature. Of note, phenylalanine has been identified as one of four novel NMR‐determined metabolites associated with incident CVD events in the FINRISK 1997 population (and validated in the SABRE and BWHHS population) using the same NMR metabolomics platform,^27^ so that its association with CVD risk may suggest common pathways to cardiac damage.

Acetate was inversely associated with HF (HR 0.81 for one SD increase, 95% CI 0.68–0.98, *P* = 0.026). This may indicate alteration in glycolysis, fatty acid and/or amino acid metabolism, dietary effects, or microbiome effects.^39^ Increased urinary acetic acid has been observed in patients with established HF.^40^ However, this observation was not replicated in FINRISK, although the differences between the cohorts must be emphasised: difference in age (24 to 74 in FINRISK vs. 70 to 82 in PROSPER); difference in event rate (3.4% at 3 years in PROSPER vs. 1.8% at 5 years in FINRISK); and likely differences in diet and lifestyle.

Measures of HDL, such as apolipoprotein A1, concentration of medium and small HDL particles and the phospholipid and cholesterol content of HDL were found to be slightly lower in those who subsequently suffered HFH vs. those who did not, although their associations were attenuated in multivariable models. Neither HDL‐cholesterol nor HDL‐cholesterol to total cholesterol ratio have been consistently related to risk of incident HF.^23^, ^32^ Our results broadly agree with other studies suggesting HDL‐cholesterol *per se* is not a useful early predictor of HF.^23^

Fatty acids provide the majority of energy substrates required by the heart.^20^ It is thought that HF may develop when fatty acids cannot be adequately utilised to meet the energy needs of the heart.^18^ We did not observe any significant associations between NMR measures of fatty acids with HFH in PROSPER, in contrast to the results from FINRISK. In FINRISK, a number of lipid measures (including ratio of MUFA, PUFA and omega‐6 fatty acid to total fatty acid) were associated with HF, some with stronger HRs than phenylalanine. Again, differences between the cohorts must be emphasised, and clearly further cohorts are needed to test our findings.

Our study has some notable strengths beyond the testing of biomarker concentrations in two cohorts. This is a comparatively large study for metabolic profiling and is made possible by high‐throughput automated NMR metabolomics.^21^ This method also allowed quantification of lipids and detailed lipoprotein analysis in addition to metabolites.^21^ Additionally, we were careful to adjust for NT‐proBNP in both cohorts, a robust predictor of incident HF. We accept some limitations. Our endpoint was based on hospitalisation for HF in PROSPER, and the decision for whether to admit a patient for HF is not standardised. Patients who developed HF without being hospitalised (milder episodes of HF) will be missed in our study, although mild episodes of HF in older age are less clinically concerning if they do not later cause hospitalisation for symptoms. There was no interaction for main effects reported here by trial treatment groups. Individuals with congestive HF (NYHA class III and IV) were excluded at baseline, however some patients with HF may be present in the non‐HF group at 6 months (the baseline for this study due to sample availability). About 25% of non‐HF individuals had a 6‐month NT‐proBNP concentration of 274 ng/L, above the 125 ng/L rule‐out value recommended in the European Society of Cardiology guidelines (negative predictive value 0.94–0.98; positive predictive value 0.44–0.57).^41^ However, suggested rule‐in values for 50–70 and >75 year olds are 900 ng/L and 1800 ng/L, respectively.^42^ Information was not available to differentiate diagnosis of HF with preserved ejection fraction (HFpEF) or HF with reduced ejection fraction (HFrEF), and associations may be different for these HF categories due to differing pathogenesis.^6^, ^19^ The definition of HF used in this study may bias prediction of HFrEF hospitalisation; however, a recent study reported little difference in number of hospitalisations for HFpEF vs. HFrEF, particularly in the Caucasian and over 75‐year age groups.^43^ Additionally, patients were not stratified by acute vs. chronic HF, ischaemic vs. non‐ischaemic HF; again associations may differ within these subgroups. Finally, samples from PROSPER and FINRISK were about 20 years old at time of NMR spectroscopy, therefore there may be degradation of some metabolic measures. However, since cases and controls were treated the same way, identified differences are robust.

In conclusion, we have demonstrated that elevated phenylalanine concentrations were reproducibly and independently associated with incident HFH. However, since addition of phenylalanine and acetate to the model did not improve HF prediction beyond established clinical predictors and NT‐proBNP, the clinical utility is likely to be low. It is possible that more detailed phenotyping available using MS metabolomics may identify more robust markers; however, this method provides only relative quantitation (generally) and is relatively expensive, limiting the number of samples that can be analysed. It is also possible that ^1^H‐NMR metabolomics of specific subtypes of HF may identify useful biomarkers for those patients. Additionally, the mechanistic pathways that lead to raised phenylalanine concentrations preceding clinical presentation with HF are of interest.

## Supporting information


**Table S1.** ^1^H‐NMR‐derived metabolite and lipoprotein measures (median and IQR) in incident HFH vs. no HFH in PROSPER (all measures shown).
**Table S2.** Hazard ratios and 95% CI for HFH vs. no HFH in PROSPER.
**Table S3.** Hazard ratios and 95% CI for incident HF vs. no HF in the FINRISK 1997 cohort.
