# Supplementary material for: Nuclear magnetic resonance‐based metabolomics identifies phenylalanine as a novel predictor of incident heart failure hospitalisation: results from PROSPER and FINRISK 1997
Source: Eur J Heart Fail. 2017 Dec 11;20(4):663–73. doi: 10.1002/ejhf.1076 (PMC5947152; doi:10.1002/ejhf.1076)
Supplement: Supplementary file 1 — Table S1. 1H‐NMR‐derived metabolite and lipoprotein measures (median and IQR) in incident HFH vs. no HFH in PROSPER (all measures shown). Table S2. Hazard ratios and 95% CI for HFH vs. no HFH in PROSPER. Table S3. Hazard ratios and 95% CI for incident HF vs. no HF in the FINRISK 1997 cohort. [file EJHF-20-663-s001.docx]

**SUPPLEMENTARY MATERIAL**

Table S1 ^1^H-NMR-derived metabolite and lipoprotein measures (median and IQR) in incident HFH vs. no HFH in PROSPER (all measures shown)

| **Metabolite or lipoprotein measure** | **No HFH (n = 5159)** | **HFH (n = 182)** | ***P*-value** |
| --- | --- | --- | --- |
| Apolipoprotein A1 (g/l) | 1.54 (1.44–1.65) | 1.49 (1.40–1.60) | **<0.001** |
| Concentration of medium HDL particles (nmol/l) | 1.77 (1.59–1.97) | 1.64 (1.48–1.88) | **<0.001** |
| Concentration of small HDL particles (nmol/l) | 4.40 (4.17–4.66) | 4.30 (3.99–4.58) | **<0.001** |
| Creatinine (μmol/l) | 71.00 (60.50–82.90) | 76.70 (64.10–96.10) | **<0.001** |
| Glycoprotein acetyls (GlycA)(mmol/l) | 1.27 (1.18–1.38) | 1.32 (1.22–1.42) | **<0.001** |
| Phenylalanine (mmol/l) | 45.10 (40.70–49.80) | 47.85 (43.30–52.40) | **<0.001** |
| Phospholipids in HDL (μmol/l) | 1.32 (1.16–1.52) | 1.25 (1.10–1.44) | **<0.001** |
| Esterified cholesterol (%) | 71.35 (70.05–72.55) | 70.94 (69.44–72.00) | **0.001** |
| Mean diameter for LDL particles (nm) | 23.70 (23.60–23.70) | 23.70 (23.60–23.70) | **0.002** |
| Total cholesterol in HDL2 (mmol/l) | 0.83 (0.68–1.03) | 0.76 (0.64–0.95) | **0.003** |
| Total cholesterol in HDL (mmol/l) | 1.31 (1.14–1.50) | 1.24 (1.10–1.44) | **0.004** |
| 3-hydroxybutyrate (mmol/l) | 0.10 (0.07–0.15) | 0.12 (0.08–0.17) | **0.011** |
| Total phospholipids (μmol/L) | 2.65 (2.41–2.90) | 2.59 (2.34–2.87) | **0.014** |
| Citrate (μmol/l) | 96.80 (81.40–112.00) | 99.85 (85.10–117.00) | 0.032 |
| Ratio of omega-3 fatty acids to total fatty acids | 3.81 (3.35–4.42) | 3.64 (3.29–4.23) | 0.033 |
| Concentration of large HDL particles (nmol/l) | 1.00 (0.76–1.29) | 0.92 (0.70–1.21) | 0.034 |
| Lactate (mmol/l) | 2.33 (1.80–3.39) | 2.44 (1.94–3.84) | 0.04 |
| Acetate (mmol/l) | 0.03 (0.02–0.03) | 0.02 (0.02–0.03) | 0.046 |
| Omega-3 fatty acids (mmol/l) | 0.41 (0.35–0.49) | 0.40 (0.33–0.47) | 0.06 |
| Pyruvate (mmol/l) | 0.11 (0.09–0.15) | 0.12 (0.09–0.16) | 0.08 |
| Histidine (mmol/l) | 0.04 (0.04–0.05) | 0.04 (0.04–0.05) | 0.1 |
| Acetoacetate (mmol/l) | 0.05 (0.04–0.07) | 0.05 (0.04–0.08) | 0.11 |
| Total phosphoglycerides (mmol/l) | 1.75 (1.56–1.97) | 1.71 (1.53–1.95) | 0.11 |
| Phosphatidylcholine and other cholines (mmol/l) | 1.77 (1.59–1.98) | 1.75 (1.56–1.95) | 0.11 |
| Docosahexaenoic acid (mmol/l) | 0.14 (0.12–0.18) | 0.14 (0.11–0.17) | 0.12 |
| Triglycerides in IDL (mmol/l) | 113.0 (94.8–136.0) | 116.0 (95.8–145.0) | 0.14 |
| Ratio of docosahexaenoic acid to total fatty acids | 1.33 (1.11–1.62) | 1.31 (1.09–1.56) | 0.2 |
| Ratio of triglycerides to phosphoglycerides | 0.62 (0.49–0.80) | 0.64 (0.53–0.82) | 0.2 |
| Isoleucine (mmol/l) | 0.04 (0.04–0.06) | 0.05 (0.04–0.06) | 0.21 |
| Concentration of very large HDL particles (nmol/l) | 380 (288–501) | 365 (282–488) | 0.3 |
| Total cholines (mmol/l) | 2.16 (1.95–2.40) | 2.12 (1.96–2.36) | 0.31 |
| Mean diameter for VLDL particles (nm) | 36.30 (35.60–37.20) | 36.20 (35.50–37.30) | 0.32 |
| Triglycerides in LDL (mmol/l) | 0.16 (0.13–0.20) | 0.17 (0.13–0.22) | 0.34 |
| Mean diameter for HDL particles (nm) | 9.94 (9.80–10.10) | 9.91 (9.78–10.10) | 0.36 |
| Alanine (mmol/l) | 0.28 (0.24–0.33) | 0.29 (0.23–0.34) | 0.38 |
| Total cholesterol in HDL3 (mmol/l) | 0.45 (0.42–0.50) | 0.45 (0.43–0.50) | 0.39 |
| Esterified cholesterol (mmol/l) | 2.93 (2.49–3.44) | 2.90 (2.44–3.40) | 0.39 |
| Leucine (mmol/l) | 0.06 (0.05–0.07) | 0.06 (0.05–0.07) | 0.39 |
| Albumin (signal area) | 0.08 (0.08–0.09) | 0.08 (0.08–0.09) | 0.41 |
| Estimated degree of unsaturation | 1.20 (1.15–1.26) | 1.19 (1.15–1.26) | 0.43 |
| Concentration of very small VLDL particles (μmol/l) | 42.40 (36.20–50.10) | 43.10 (36.90–51.90) | 0.44 |
| Valine (mmol/l) | 0.14 (0.12–0.16) | 0.14 (0.12–0.16) | 0.46 |
| Ratio of polyunsaturated fatty acids to total fatty acids | 36.20 (33.50–38.80) | 36.20 (33.50–38.60) | 0.48 |
| Polyunsaturated fatty acids (mmol/l) | 3.80 (3.34–4.38) | 3.80 (3.35–4.27) | 0.48 |
| Ratio of monounsaturated fatty acids to total fatty acids | 24.90 (22.40–27.70) | 25.30 (22.40–28.20) | 0.55 |
| Glutamine (mmol/l) | 0.42 (0.38–0.45) | 0.42 (0.38–0.46) | 0.57 |
| Serum total cholesterol (mmol/l) | 4.08 (3.48–4.83) | 4.09 (3.48–4.77) | 0.58 |
| Concentration of large VLDL particles (nmol/l) | 3.69 (1.94–6.24) | 3.62 (1.89–6.20) | 0.59 |
| Omega-6 fatty acids (mmol/l) | 3.38 (2.94–3.91) | 3.36 (2.98–3.81) | 0.64 |
| Concentration of IDL particles (nmol/l) | 97.10 (80.50–118.00) | 100.50 (79.80–120.00) | 0.65 |
| Sphingomyelins (mmol/l) | 0.47 (0.41–0.53) | 0.47 (0.41–0.53) | 0.66 |
| Ratio of linoleic acid to total fatty acids | 25.70 (23.10–28.30) | 25.40 (22.90–28.20) | 0.67 |
| Glucose (mmol/l) | 3.28 (2.56–3.82) | 3.27 (2.30–3.77) | 0.69 |
| Concentration of very large VLDL particles (nmol/l) | 0.47 (0.17–0.92) | 0.44 (0.17–0.94) | 0.7 |
| Linoleic acid (mmol/l) | 2.70 (2.31–3.20) | 2.70 (2.31–3.18) | 0.72 |
| Phospholipids in LDL (μmol/l) | 0.56 (0.48–0.67) | 0.57 (0.48–0.66) | 0.72 |
| Saturated fatty acids (mmol/l) | 4.13 (3.57–4.77) | 4.12 (3.61–4.74) | 0.73 |
| Free cholesterol (mmol/l) | 1.17 (0.99–1.40) | 1.17 (1.03–1.42) | 0.73 |
| Remnant cholesterol (non-HDL-, non-LDL-cholesterol) (mmol/l) | 1.43 (1.15–1.76) | 1.44 (1.18–1.77) | 0.75 |
| Ratio of omega-6 fatty acids to total fatty acids | 32.10 (29.60–34.70) | 32.00 (29.60–34.70) | 0.76 |
| Total fatty acids (mmol/l) | 10.60 (9.28–12.20) | 10.40 (9.27–12.10) | 0.76 |
| Tyrosine (mmol/l) | 0.06 (0.05–0.06) | 0.06 (0.05–0.07) | 0.76 |
| Total cholesterol in VLDL (mmol/l) | 0.80 (0.64–1.01) | 0.81 (0.66–1.00) | 0.77 |
| Concentration of small LDL particles (nmol/l) | 138.00 (111.00–171.00) | 138.50 (108.00–172.00) | 0.78 |
| Concentration of large LDL particles (μmol/l) | 150.00 (120.00–187.00) | 155.00 (118.00–188.00) | 0.8 |
| Concentration of medium VLDL particles (μmol/l) | 15.30 (10.50–21.90) | 15.40 (10.50–22.10) | 0.8 |
| Concentration of medium LDL particles (nmol/l) | 117.00 (91.80–149.00) | 121.00 (91.00–151.00) | 0.84 |
| Concentration of small VLDL particles (μmol/l) | 32.60 (26.40–40.20) | 32.25 (26.10–42.10) | 0.85 |
| Triglycerides in VLDL (mmol/l) | 0.77 (0.53–1.10) | 0.77 (0.52–1.13) | 0.85 |
| Monounsaturated fatty acids; 16:1, 18:1 (mmol/l) | 2.62 (2.17–3.21) | 2.63 (2.06–3.28) | 0.86 |
| Non-HDL-cholesterol (mmol/l) | 2.75 (2.18–3.46) | 2.82 (2.21–3.45) | 0.87 |
| Apolipoprotein B (g/l) | 0.90 (0.76–1.07) | 0.91 (0.76–1.10) | 0.88 |
| Ratio of saturated fatty acids to total fatty acids | 38.90 (36.80–40.80) | 38.90 (37.00–40.90) | 0.9 |
| Phospholipids in IDL (μmol/l) | 253.00 (209.00–306.00) | 254.50 (204.00–306.00) | 0.92 |
| Triglycerides in HDL (mmol/l) | 0.13 (0.11–0.16) | 0.13 (0.11–0.17) | 0.93 |
| Serum total triglycerides (mmol/l) | 1.19 (0.90–1.57) | 1.19 (0.88–1.62) | 0.94 |
| Total cholesterol in IDL (μmol/l) | 616.00 (497.00–765.00) | 622.50 (488.00–768.00) | 0.95 |
| Concentration of chylomicrons and extremely large VLDL particles (nmol/l) | 0.09 (0.04–0.17) | 0.09 (0.04–0.17) | 0.96 |
| Phospholipids in VLDL (μmol/l) | 0.45 (0.35–0.58) | 0.45 (0.34–0.60) | 0.98 |
| Total cholesterol in LDL (mmol/l) | 1.33 (1.01–1.72) | 1.37 (0.98–1.70) | 0.99 |

The median and IQR for all included metabolite, lipid and lipoprotein measures are shown in HFH versus no HFH. Measures shown in order of ascending p-value; those ≤0.014 are shown in bold, notionally significant after correcting for false discovery using the Benjamini and Hochberg method and a false discovery rate of 0.1.

Table S2 Hazard ratios and 95% CI for HFH vs. no HFH in PROSPER

| **Metabolite or lipoprotein measure** | **Hazard Ratio (95% CI)** | ***P*-value** |
| --- | --- | --- |
| Phenylalanine | 1.29 (1.10–1.53) | **0.002** |
| Acetate | 0.81 (0.68–0.98) | **0.0257** |
| Creatinine | 1.28 (0.99–1.66) | 0.0598 |
| Concentration of medium HDL particles | 0.86 (0.72–1.01) | 0.0661 |
| Glycoprotein acetyls (GlycA) | 1.14 (0.98–1.33) | 0.0975 |
| Ratio of omega-3 fatty acids to total fatty acids | 0.88 (0.75–1.03) | 0.1203 |
| Tyrosine | 1.12 (0.96–1.31) | 0.1389 |
| Omega-3 fatty acids | 0.88 (0.74–1.04) | 0.1429 |
| Total cholesterol in HDL2 | 0.89 (0.75–1.05) | 0.1521 |
| Total cholesterol in HDL | 0.88 (0.74–1.05) | 0.1536 |
| Apolipoprotein AI | 0.88 (0.74–1.05) | 0.1569 |
| Concentration of large HDL particles | 0.90 (0.76–1.05) | 0.1722 |
| Phospholipids in HDL | 0.89 (0.75–1.06) | 0.1803 |
| Leucine | 1.12 (0.94–1.34) | 0.2008 |
| 3-hydroxybutyrate | 1.10 (0.95–1.27) | 0.2242 |
| Albumin | 0.92 (0.80–1.07) | 0.2725 |
| Isoleucine | 1.10 (0.92–1.32) | 0.2841 |
| Total phospholipids | 0.91 (0.76–1.08) | 0.2903 |
| Concentration of small HDL particles | 0.92 (0.79–1.09) | 0.3427 |
| Glucose | 1.08 (0.92–1.27) | 0.3481 |
| Esterified cholesterol | 0.92 (0.77–1.11) | 0.3739 |
| Mean diameter for LDL particles | 1.07 (0.92–1.23) | 0.376 |
| Ratio of polyunsaturated fatty acids to total fatty acids | 0.94 (0.80–1.09) | 0.3989 |
| Ratio of triglycerides to phosphoglycerides | 1.07 (0.91–1.26) | 0.4014 |
| Valine | 1.08 (0.90–1.28) | 0.4091 |
| Serum total cholesterol | 0.92 (0.76–1.12) | 0.4203 |
| Lactate | 1.08 (0.89–1.32) | 0.433 |
| Alanine | 1.06 (0.91–1.24) | 0.455 |
| Mean diameter for HDL particles | 0.94 (0.79–1.11) | 0.4648 |
| Esterified cholesterol | 0.95 (0.82–1.10) | 0.4706 |
| Phospholipids in IDL | 0.94 (0.77–1.13) | 0.4933 |
| Total cholesterol in IDL | 0.94 (0.77–1.13) | 0.496 |
| Polyunsaturated fatty acids | 0.94 (0.79–1.12) | 0.4971 |
| Docosahexaenoic acid | 0.95 (0.81–1.11) | 0.5024 |
| Concentration of large VLDL particles | 0.95 (0.81–1.11) | 0.5061 |
| Histidine | 0.95 (0.83–1.10) | 0.5101 |
| Ratio of docosahexaenoic acid to total fatty acid | 0.95 (0.82–1.11) | 0.5273 |
| Concentration of very large HDL particles | 0.95 (0.80–1.12) | 0.5276 |
| Free cholesterol | 0.94 (0.77–1.15) | 0.5699 |
| Concentration of IDL particles | 0.95 (0.78–1.15) | 0.5836 |
| Non-HDL-cholesterol | 0.95 (0.79–1.15) | 0.6184 |
| Total cholines | 0.96 (0.81–1.14) | 0.6279 |
| Ratio of saturated fatty acids to total fatty acids | 1.04 (0.89–1.22) | 0.6316 |
| Ratio of omega-6 fatty acids to total fatty acids | 0.96 (0.82–1.13) | 0.6376 |
| Concentration of very small VLDL particles | 0.96 (0.79–1.15) | 0.6399 |
| Estimated degree of unsaturation | 0.96 (0.82–1.13) | 0.644 |
| Total phosphoglycerides | 0.96 (0.81–1.14) | 0.6477 |
| Omega-6 fatty acids | 0.96 (0.81–1.14) | 0.6522 |
| Remnant cholesterol (non-HDL, non-LDL cholesterol) | 0.96 (0.79–1.16) | 0.6538 |
| Concentration of small LDL particles | 0.96 (0.81–1.14) | 0.6544 |
| Phosphatidylcholine and other cholines | 0.96 (0.81–1.14) | 0.6617 |
| Total cholesterol in LDL | 0.96 (0.80–1.15) | 0.6653 |
| Acetoacetate | 0.97 (0.82–1.13) | 0.6672 |
| Mean diameter for VLDL particles | 1.04 (0.88–1.22) | 0.6678 |
| Linoleic acid | 0.96 (0.81–1.14) | 0.6743 |
| Ratio of linoleic acid to total fatty acids | 0.97 (0.83–1.13) | 0.6973 |
| Apolipoprotein B | 0.96 (0.80–1.16) | 0.7045 |
| Pyruvate | 1.03 (0.89–1.19) | 0.7132 |
| Concentration of large LDL particles | 0.97 (0.80–1.17) | 0.7186 |
| Concentration of chylomicrons and extremely large VLDL particles | 0.97 (0.83–1.14) | 0.7286 |
| Total cholesterol in HDL3 | 0.97 (0.81–1.16) | 0.7428 |
| Total cholesterol in VLDL | 0.97 (0.81–1.17) | 0.7832 |
| Triglycerides in IDL | 1.02 (0.86–1.21) | 0.7908 |
| Citrate | 1.02 (0.87–1.19) | 0.7992 |
| Serum total triglycerides | 1.02 (0.86–1.21) | 0.8121 |
| Triglycerides in VLDL | 1.02 (0.86–1.20) | 0.837 |
| Glutamine | 1.01 (0.88–1.17) | 0.8393 |
| Concentration of medium LDL particles | 0.98 (0.82–1.17) | 0.8443 |
| Phospholipids in LDL | 0.98 (0.83–1.16) | 0.8448 |
| Monounsaturated fatty acids; 16:1, 18:1 | 0.99 (0.83–1.17) | 0.8681 |
| Triglycerides in LDL | 1.01 (0.85–1.20) | 0.8882 |
| Total fatty acids | 0.99 (0.83–1.18) | 0.9009 |
| Ratio of monounsaturated fatty acids to total fatty acids | 0.99 (0.84–1.17) | 0.9103 |
| Sphingomyelins | 0.99 (0.84–1.16) | 0.9128 |
| Concentration of small VLDL particles | 0.99 (0.84–1.18) | 0.9368 |
| Saturated fatty acids | 1.01 (0.85–1.19) | 0.9468 |
| Concentration of medium VLDL particles | 1.00 (0.85–1.19) | 0.9607 |
| Triglycerides in HDL | 1.00 (0.84–1.18) | 0.9854 |
| Phospholipids in VLDL | 1.00 (0.84–1.19) | 0.9888 |
| Concentration of very large VLDL particles | 1.00 (0.86–1.16) | 0.9977 |

Hazard ratios for incident HFH events in PROSPER during 2.7 years of follow-up. Associations were adjusted for treatment group, age, sex, smoking status, country, BMI, MI, SBP, DBP, CABG, PTCA, TIA, stroke, angina, claudication, PVD, diabetes, eGFR, NT-proBNP concentration (6 month) and treatment with ACE inhibitors, beta-blockers, calcium channel blockers, anti-arrhythmics and diuretics (note all medications are as recorded at baseline (0 month)). Abbreviation list as for Table 1. All included NMR measures are shown in order of ascending p-value. Those with p<0.05 are shown in bold.

Table S3 Hazard ratios and 95% CI for incident HF vs. no HF in the FINRISK 1997 cohort

| **Metabolite** | **Hazard Ratio (95% CI)** | ***P*-value** |
| --- | --- | --- |
| Ratio of monounsaturated fatty acids to total fatty acids | 1.42 (1.21–1.66) | **1.50^-05^** |
| Glucose | 1.21 (1.09–1.35) | **0.00059** |
| Ratio of omega-6 fatty acids to total fatty acids | 0.756 (0.642–0.89) | **0.00076** |
| Pyruvate | 1.28 (1.11–1.47) | **0.0008** |
| Ratio of polyunsaturated fatty acids to total fatty acids | 0.78 (0.666–0.912) | **0.0018** |
| Total cholesterol in HDL2 | 0.78 (0.666–0.912) | **0.0018** |
| Triglycerides in HDL | 1.27 (1.09–1.49) | **0.0028** |
| Ratio of linoleic acid to total fatty acids | 0.782 (0.666–0.919) | **0.0028** |
| Monounsaturated fatty acids; 16:1, 18:1 | 1.26 (1.06–1.5) | **0.0088** |
| Isoleucine | 1.26 (1.05–1.5) | **0.012** |
| Lactate | 1.23 (1.04–1.44) | **0.014** |
| Serum total triglycerides | 1.26 (1.05–1.52) | **0.015** |
| Mean diameter for VLDL particles | 1.23 (1.03–1.46) | **0.02** |
| Phenylalanine | 1.23 (1.03–1.48) | **0.023** |
| Alanine | 1.22 (1.02–1.45) | **0.025** |
| Ratio of triglycerides to phosphoglycerides | 1.18 (1.02–1.37) | **0.026** |
| Glycoprotein acetyls (GlycA) | 1.21 (1.02–1.44) | **0.033** |
| Tyrosine | 1.23 (1.02–1.49) | **0.033** |
| Triglycerides in LDL | 1.15 (1.01–1.32) | **0.041** |
| 3-hydroxybutyrate | 0.811 (0.663–0.992) | **0.041** |
| Triglycerides in VLDL | 1.18 (1–1.38) | **0.044** |
| Total cholesterol in LDL | 0.831(0.694–0.996) | **0.045** |
| Triglycerides in IDL | 1.16 (1–1.34) | **0.047** |
| Phospholipids in IDL | 1.16 (1–1.34) | **0.047** |
| Concentration of small HDL particles | 1.16 (0.988–1.37) | 0.07 |
| Concentration of small LDL particles | 1.16 (0.988–1.37) | 0.07 |
| Degree of fatty acid saturation | 0.863 (0.732–1.02) | 0.08 |
| Esterified cholesterol | 0.851 (0.708–1.02) | 0.087 |
| Serum total cholesterol | 0.861 (0.714–1.04) | 0.12 |
| Leucine | 1.15 (0.96–1.38) | 0.13 |
| Concentration of large VLDL particles | 1.1 (0.968–1.24) | 0.15 |
| Creatinine | 1.15 (0.951–1.4) | 0.15 |
| Glutamine | 0.884 (0.743–1.05) | 0.16 |
| Concentration of medium VLDL particles | 1.11 (0.959–1.28) | 0.17 |
| Total cholesterol in HDL3 | 1.11 (0.954–1.29) | 0.18 |
| Total fatty acids | 1.13 (0.946–1.34) | 0.18 |
| Concentration of very large VLDL particles | 1.08 (0.962–1.22) | 0.19 |
| Saturated fatty acids | 1.1 (0.95–1.27) | 0.2 |
| Phospholipids in LDL | 0.884 (0.733–1.07) | 0.2 |
| Valine | 1.14 (0.928–1.4) | 0.21 |
| Non-HDL-cholesterol | 0.885 (0.729–1.07) | 0.21 |
| Total phosphoglycerides | 1.12 (0.935–1.34) | 0.22 |
| Concentration of small VLDL particles | 1.1 (0.937–1.29) | 0.24 |
| Free cholesterol | 0.893 (0.738–1.08) | 0.25 |
| Total cholesterol in IDL | 0.893 (0.738–1.08) | 0.25 |
| Mean diameter for HDL particles | 0.901 (0.752–1.08) | 0.26 |
| Glycine (NA in PROSPER as EDTA plasma) | 1.11 (0.915–1.35) | 0.29 |
| Phosphatidylcholine and other cholines | 1.1 (0.917–1.32) | 0.3 |
| Phospholipids in VLDL | 1.08 (0.928–1.25) | 0.32 |
| Glycerol (NA in PROSPER as EDTA plasma) | 1.1 (0.904–1.34) | 0.34 |
| Sphingomyelins | 0.916 (0.764–1.1) | 0.35 |
| Concentration of IDL particles | 0.913 (0.753–1.11) | 0.36 |
| Apolipoprotein A1 | 0.926 (0.775–1.11) | 0.4 |
| Total cholesterol in HDL | 0.929 (0.782–1.1) | 0.4 |
| Acetoacetate | 0.933 (0.785–1.11) | 0.43 |
| Total choline | 1.07 (0.894–1.28) | 0.46 |
| Phospholipids in HDL | 1.07 (0.891–1.29) | 0.46 |
| Ratio of omega-3 fatty acids to total fatty acids | 0.945 (0.8–1.12) | 0.5 |
| Concentration of chylomicrons and extremely large VLDL particles | 1.04 (0.926–1.17) | 0.5 |
| Linoleic acid | 0.95 (0.815–1.11) | 0.51 |
| Apolipoprotein B | 0.944 (0.777–1.15) | 0.56 |
| Albumin | 0.947 (0.787–1.14) | 0.56 |
| Total phospholipids | 1.05 (0.878–1.27) | 0.57 |
| Total cholesterol in VLDL | 1.06 (0.861–1.3) | 0.59 |
| Remnant cholesterol (non-HDL, non-LDL cholesterol) | 0.952 (0.789–1.15) | 0.6 |
| Docosahexaenoic acid | 1.04 (0.893–1.22) | 0.6 |
| Concentration of very large HDL particles | 0.953 (0.787–1.15) | 0.62 |
| Omega-6 fatty acids | 0.96 (0.814–1.13) | 0.63 |
| Histidine | 0.96 (0.804–1.15) | 0.65 |
| Concentration of medium HDL particles | 1.04 (0.871–1.23) | 0.69 |
| Concentration of medium LDL particles | 1.04 (0.871–1.23) | 0.69 |
| Citrate | 0.966 (0.814–1.15) | 0.7 |
| Polyunsaturated fatty acids | 0.968 (0.817–1.15) | 0.7 |
| Ratio of docosahexaenoic acid to total fatty acid | 0.971 (0.826–1.14) | 0.72 |
| Mean diameter for LDL particles | 1.03 (0.867–1.22) | 0.76 |
| Ratio of saturated fatty acids to total fatty acids | 1.02 (0.877–1.2) | 0.76 |
| Concentration of large HDL particles | 1.02 (0.839–1.25) | 0.81 |
| Concentration of large LDL particles | 1.02 (0.839–1.25) | 0.81 |
| Concentration of very small VLDL particles | 1.01 (0.848–1.2) | 0.91 |
| Omega-3 fatty acids | 1 (0.834–1.21) | 0.97 |
| Acetate | 1 (0.842–1.19) | 0.99 |

HRs in the FINRISK 1997 cohort, sorted by p-value (n=7,330; 133 events during 5 year follow-up). Adjusted for sex, SBP, DBP, smoking, diabetes, lipid lowering therapy, BP lowering therapy, prevalent CVD, eGFR (based on NMR measured creatinine) and NT-proBNP.
